# The North-Eastern Europe and Northern Asia isotopic dataset of bioarchaeological samples (NEENA)

**DOI:** 10.1038/s41597-025-06477-5

**Published:** 2026-01-14

**Authors:** Vera Haponava, Catriona Pickard, Ricardo Fernandes

**Affiliations:** 1 School of History, Classics and Archaeology, University of Edinburgh, Edinburgh, UK; 2 Max Planck Institute of Geoanthropology, Jena, Germany; 3 Department of Bioarchaeology, Faculty of Archaeology, University of Warsaw, Warszawa, 00-927 Poland; 4 Climate Change and History Research Initiative, Princeton University, Princeton, NJ 08544 USA

**Keywords:** Archaeology, Biological anthropology, Stable isotope analysis

## Abstract

The North-Eastern Europe and Northern Asia open-access dataset (NEENA) is a compilation of over 18,700 isotopic measurements (δ^13^C, δ^15^N, δ^34^S, δ^18^O, ^87^Sr/^86^Sr), predominantly from archaeological human, animal, and plant samples originating from more than 750 sites ranging geographically from the Baltic and Eastern Europe to North-Central Asia and dating between 70,000 years BP and modern times. For each isotope record included in the dataset, information relating to the taxonomic categorisation of the sampled material (e.g., animal and plant species or genus names), the sample type (e.g., bone, dentine, enamel) and contextual, chronological, provenance (i.e., site location and country), and laboratory details are provided where available from original publications. The NEENA dataset can be used to conduct comparative studies of palaeodiet, spatial mobility, paleo-environmental conditions, organic remains preservation, and radiocarbon reservoir effects. NEENA is available in an open-access format via the Pandora data platform.

## Background & Summary

Conventionally, studies of past diet in archaeological populations relied on indirect evidence such as written sources or the analysis of faunal assemblages and plant remains, which at best reflect population level consumption practices. Likewise, it is well known that mobility established from the presence of ‘non-local’ or ‘exotic’ material culture remains does not necessarily reflect the provenance, nor the movement, of people^1^. The ability to obtain meaningful data through the direct analysis of individuals enhances the study of not only well-contextualized remains but also those recovered from uncertain or mixed contexts, as well as decontextualized materials^2^. Isotope ratio measurements constitute direct evidence of an individual’s behaviour or life history, and are therefore a useful addition to other lines of evidence (e.g., material culture remains, and bioarchaeological and biomolecular analyses): most importantly, however, analysis of isotope data is one of the most widely employed approaches for directly investigating socioculturally informed behaviours such as the sex- and age-related differences in diets^3^.

Stable isotopes were first deployed for archaeological research in the 1970s in the works of van der Merwe and Vogel^4^, and DeNiro and Epstein^5,6^. These studies demonstrated the utility of stable carbon isotope ratios (δ^13^C) for elucidating palaeodiets (e.g., distinguishing between the direct consumption of C_3_ and C_4_ plants or of animals fed on these). Shortly thereafter, stable nitrogen isotopes (δ^15^N) were demonstrated to reflect the relative trophic position of a consumer^7^, and co-analyses of δ^13^C and δ^15^N were found to be useful in distinguishing between marine and terrestrial food consumption^8–10^. Over the past five decades the application of stable isotope analysis has expanded considerably and now incorporates the isotopic ratios of a wider range of elements (e.g., hydrogen, lead, oxygen, sulfur, strontium and zinc), and a larger suite of sample types and compounds^2,3,11,12^. The range of research questions investigated through stable isotope analysis has also expanded substantially (e.g., modelling of human breastfeeding and weaning, materials provenance, agricultural practices, cooking, residence, and mobility and migration)^2,3^.

**Fig. 1:**
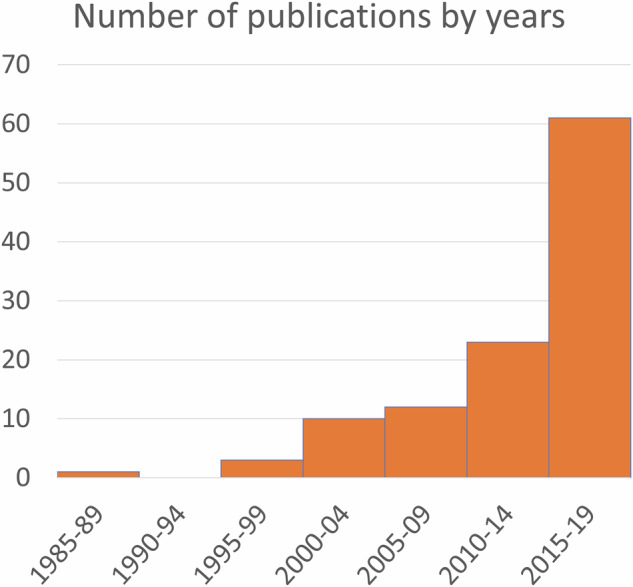
Number of publications in NEENA by half decade, excluding the years 2020–2022 before the start of data collection in 2023.

With reductions in sample processing and analytical costs, an expansion in the availability of equipment and stable isotope laboratories, as well as the broadening of the knowledge base of possible applications, archaeological studies incorporating stable isotope analysis have increased exponentially since their development in the 1970s^13^ (Fig. 1). As demonstrated in the compiled measurements presented here, studies have generally focused on one or a small number of sites, and although there are exceptions, typically, published isotopic datasets are small, ranging from a single isotopic measurement to several hundred measurements^14–19^. With the growing corpus of studies and the large volume of data being generated by numerous separate studies, the need for a ‘Big Data’ approach has become apparent^20^. Synthesizing data enables large-scale analysis of isotopic variation over time and space, uncovers diachronic trends, and exposes gaps in knowledge^21^, thus informing future research. Initiatives like IsoMemo^22^, IsoBank^20^, and IsoArcH^23^ were established to facilitate the collection, standardisation, and sharing of data following FAIR and CARE principles^24^. Such enterprises bring enormous benefits to the ever-expanding range of isotopic research. Environmental and food source isotope ranges are neither geospatially nor temporally universal but vary regionally and chronologically^2,25,26^. Interpretation and modelling of, for example, past diet or mobility from stable isotope values requires the use of appropriate comparative (or baseline) data^2,3^. Global datasets provide such data in an accessible and searchable format, facilitating the querying and filtering of data to identify relevant archaeological sites, sample types, archaeological and geographic context, and laboratory information.

Multiple databases that collate regional and/or temporal stable isotope measurements have been published, and these cover Medieval^27^ and Classic Antiquity Europe^28^, the Mediterranean^21^, Japanese Islands^29^, Africa^30^, America^31^, and North-Central Asia^32^. Compilation of European isotope measurements have, however, focused on Western, Southern^21,28^, and Northern Europe^33^, and have been limited in chronological scope^27,28^ or sample type^33^, highlighting a gap in the representation of Eastern Europe and the Baltic. Though a part of the published isotope measurements for this region was included in other compilations^32,33^, these projects used different selection and/or recording criteria. Such compilations were included along with augmented metadata in a new dataset (see below) to aggregate a consistent dataset for the whole region, which would also fit within the group of initiatives aiming to collect isotopic data covering all the regions of the world. Availability of multiple databases, including those covering the same regions or timespans, facilitates the findability of data, while application of different recording rules makes various databases more suitable for specific research needs.

Chronologically, stable isotope research in North-Eastern Europe and Northern Asia has spanned from the Middle Palaeolithic with studies looking at the diets of woolly rhinoceros and Neanderthals^34,35^, through the start of the Neolithic with animal domestication and transmission of millet cultivation in Eurasia^36,37^, to the diet of Napoleon’s Army in the 19th century CE^38^. With its diverse natural environments and human lifeways, the region was an important economic and cultural link between East and West^32^, and home to Iron Age and Medieval nomadic empires^32^, and at one time held the largest state in Europe, the Grand Duchy of Lithuania of the 15th century CE^39,40^. Among many promising directions of enquiry, stable isotopes have been deployed here to investigate: the transition from the Mesolithic to the Neolithic^41,42^; regional husbandry and herding strategies^43–47^; aspects of unique animal burials^18,48^, as well as human boat burials^49,50^; fish consumption in the Neolithic^12,51–54^; diet in medieval urban and rural settlements^11,19,55^; mobility of hunter-gatherers^56^, pastoralists^57,58^ and agriculturalists^59,60^; and human dietary freshwater radiocarbon reservoir effects^15,61–63^.

The North-Eastern Europe and Northern Asia dataset (NEENA)^64^ collates all stable isotope data from the Baltic region, Eastern Europe, and North-Central Asia published in English and in local languages, such as Russian and Polish. Each measurement record is complemented with contextual and chronological information, geographical coordinates, age and sex assessment of human individuals, taxonomic categorisation of animals and plants, and laboratory information and quality controls - all of which enables a wide range of research directions. To facilitate additional research on the samples and sites included in the dataset, bibliographical information is provided therein for each record.

**Fig. 2:**
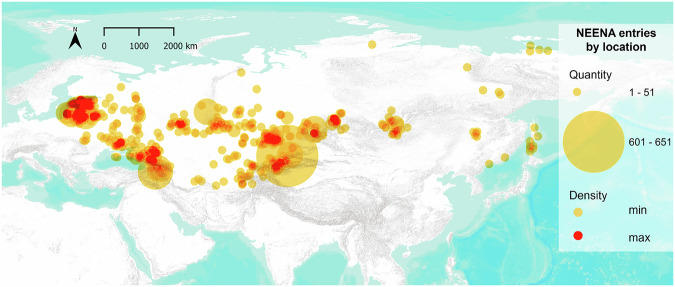
Spatial distribution of sites included in the dataset. The size of the circle reflects the number of entries originating from a specific location, while colour intensity reflects their density. The map was created in QGIS 3.34 software using ESRI Terrain basemap.

The geographic region covered by the dataset spans from the Baltic Sea in the west, the Black and Caspian Seas to the south, and the Bering sea in the east (i.e., the Baltic, Eastern Europe, and Northern and Central Asia) (Fig. 2). It includes data from territories represented by the modern countries of Armenia, Azerbaijan, Belarus, Estonia, Georgia, Kazakhstan, Kyrgyzstan, Latvia, Lithuania, Russia, Tajikistan, Turkmenistan, Ukraine, and Uzbekistan. From a temporal perspective, the dataset represents a broad timespan from the Middle Palaeolithic to the present (c. 70,000 years ago to 2023 CE).

## Methods

A search for published archaeological studies with new isotopic data was conducted in 2023 in two databases: Web of Science (https://www.webofscience.com/) and Scopus (https://www.scopus.com/). The queries were posed in English and included isotope related keywords (e.g., isotop*, carbon, nitrogen, and ^87^Sr/^86^Sr) and those delimiting the target geographic region (country names like Kazakhstan*, Ukrain*, and Lithuania*). Since the attempted searches in Russian, one of the most commonly used languages in the region, yielded few results in these two databases, an additional search was conducted in Google Scholar using Russian keywords (e.g., археология, стабильные изотопы).

Scopus query: (TITLE (isotop* OR carbon OR nitrogen OR strontium OR “87Sr/86Sr” OR oxygen OR sulfur OR sulphur OR diet* OR mobility OR migration OR provenance)) AND (TITLE-ABS-KEY (isotop* AND (human OR animal OR plant))) AND (TITLE-ABS-KEY (Kazakhstan* OR Kyrgyzstan* OR Tajikistan* OR Turkmen* OR Uzbek* OR Belarus* OR Moldova* OR Russia* OR Ukrain* OR Estonia* OR Latvia* OR Lithuania* OR Armenia* OR Azerbaijan* OR Georgia*)) AND (LIMIT-TO (SUBJAREA,“ARTS”))

Web of Science query: ((TI = (isotop* OR carbon OR nitrogen OR strontium OR “87Sr/86Sr” OR oxygen OR sulfur OR sulphur OR diet* OR mobility OR migration OR provenance)) AND AB = (isotop* AND (human OR animal OR plant))) AND AB = (Kazakhstan* OR Kyrgyzstan* OR Tajikistan* OR Turkmen* OR Uzbek* OR Belarus* OR Moldova* OR Russia* OR Ukrain* OR Estonia* OR Latvia* OR Lithuania* OR Armenia* OR Azerbaijan* OR Georgia*)

Google Scholar query: археология AND стабильные изотопы AND (питание OR диета OR рацион OR мобильность OR миграция)

The keyword search yielded 164 results in Web of Sciences, of which 108 appeared to be related to archaeology, and 84 results in Scopus. Articles that did not fit the search criteria were then filtered out, usually by scrutinising the title and abstract, or where the relevance of the article was not evident from the abstract by considering the full text. From the initial search, 67 studies were added to the dataset. Publications were excluded where they:

- did not produce new data or did not publish raw data;
- analysed materials or topics that were outside the scope of NEENA (e.g., soil, water, modern plants or animals; drugs abuse and counterfeit wines, nuclear power plant sites, troposphere studies, plant biology, paleontology, geology);
- did not use samples from the target region (e.g., mentioned the state of Georgia in the US instead of the country of Georgia);
- were duplicate articles that overlapped between the databases.

Since the query in Google Scholar was less specific than in the other two databases, out of the 454 results it produced, most were excluded based on the title, and the majority of the ones that were inspected did not provide isotope data or fell out of the scope of the analysed materials or the provenance of the samples were not within employed criteria. Twelve publications were included in the dataset.

References cited in the initially identified studies were also examined and additional relevant articles were sourced from them (28 articles). Likewise, several published stable isotope databases were sourced, in particular NCA^32^ (26 articles), and dIANA^33^ (8 articles).

Some of the variables entered into the dataset were standardised for consistency (e.g., sex, age category, latitude and longitude, and min and max dates), others were entered in free-text format (e.g., context ID, site description, additional age description, and probable local power). The ‘Metadata’ sheet of the dataset files contains details on whether and how the values for a specific column were standardised, as well as whether they were treated as mandatory or optional entries.

Where some data (e.g., site location or date) were not given in the source article along with isotopic values, or it was not clear from the source publication how to transform its data into the format used in NEENA, an additional literature search was conducted to establish the relevant details for the site, period, or region in question. Commonly, this would include adding missing site information, location data, or the date range, and also cross-checking whether analysis of a sample or an individual in one publication was duplicated in other publications. For example, where only a broad time period (e.g., Late Neolithic) was provided for a site in a source publication, an additional literature search was conducted to establish a numeric time range for this period at a given site or in a specific region. The general principle adopted when populating the dataset was to always select the most precise data available while prioritising accuracy and avoiding additional assumptions. For example, a hierarchical approach was followed for chronological assignments - direct dates of a sample were reported where available; alternatively, the date of the burial context, the site, or the culture was successively used. If reviewed literature suggested that the direct (radiocarbon) date was inaccurate, an associated date such as that of the burial context or site was included. Where a publication reported the age of an individual as ‘juvenile’ without specifying the age range of this category, no numerical age range was assumed and the individual was reported as ‘non-adult’.

Records sourced from other compiled datasets were, on the whole, reported as presented; however, modifications to some entries were made in the following instances: (i) where they did not contain fields considered mandatory for NEENA (for example, min and max date ranges, and uncertainty radius for locations); (ii) to ensure consistency in the presentation of the data with the format adopted in NEENA; and (iii) where these entries did not meet the validation process described below.

Quality-checks on the collected dataset were undertaken, and included the following steps:

- Rechecking the source literature where the numeric data fell out of expected ranges or showed extreme values (for example, δ^15^N >20‰ or <0‰, or max age >100);
- For fields that are categorical (i.e., not free-form text entries), a check that all values match the predefined list of categories;
- Mandatory variables must be non-empty;
- Clear rules for data entry (e.g., no sex assigned to non-adults; age category to fit the numeric age range; uncertainty radius = 0 where site location is ‘exact’ and >0 where the location is not exact; site locations to be within specified modern countries; max year ≥ min year; all general periods to fit the numeric date range; number of samples not empty if a corresponding isotopic measurement is not empty);
- Consistency check (e.g., a single site to have the same coordinates throughout the table and different sites to have distinct coordinates; references in Harvard style, a single publication to have the same link, Digital Object Identifier (DOI), and publication date throughout; the same environment, trophic level, and family assigned to the same animal or plant genus and species);
- Unique fields such as sample ID, individual ID to be indeed unique and not to be duplicated between source publications.

The validated data still includes samples with extreme values and instances where connected variables are non-standard. Here are some examples: a sample that failed the collagen quality checks may have δ^15^N and δ^13^C outside the expected range^65^; some non-adults are assigned a sex, determined through aDNA analysis^65^; bears are typically recorded as omnivores, however, where a source publication highlighted that sampled bears had carnivorous behaviour, this would be reported in the dataset entry^66^. Deposition and maintenance of datasets in Pandora is an ongoing and collaborative effort, and further validation of the dataset by its users and correction of inconsistencies and enhancing the quality of the dataset is welcomed. Expansion of the dataset with newly published isotopic data pertaining to the region is planned.

## Data Records

The dataset is deposited within the ‘NEENA Stable Isotopes v.1’ repository^64^ with 10.48493/jgfb-5309, within the ‘NEENA’ data community (https://pandoradata.earth/organization/neena) of the Pandora data platform (https://pandoradata.earth/). The NEENA community is also part of the IsoMemo network (https://pandoradata.earth/group/isomemo-group). The ‘NEENA Stable Isotopes v.1’ repository offers compiled isotopic data as Excel [filename.xlsx] and CSV [filename.csv] files. The full isotopic dataset is separated into files for human (5,145 entries), animal (4,609 entries), and plant (77 entries) records, and includes a separate file describing the metadata. In total, 149 studies (journal articles, conference papers, book chapters, PhD theses) were included in the dataset, the majority of them published in English and several in Russian and Polish. Most of the records compiled in NEENA originate from Russia (33%), followed by Kazakhstan (19%) and Lithuania (13%). As can be observed from the spatial distribution of site locations compiled in NEENA (Fig. 2) the south of Central Asia and Georgia in the Caucasus region form a major gap in the coverage of isoscapes, together with parts of Eastern Europe (Moldova, Belarus).

Data community members in the Pandora platform may create and edit datasets, link them together, and assign DOIs to deposit materials. This allows individuals and groups of researchers to share their isotopic data while easily tracking and acknowledging the work on their production, collection, and aggregation, as well as to collaborate with other members. The NEENA data community compiles all types of bioarchaeological data from East Europe and North Asia and welcomes new members and contributions.

**Fig. 3:**
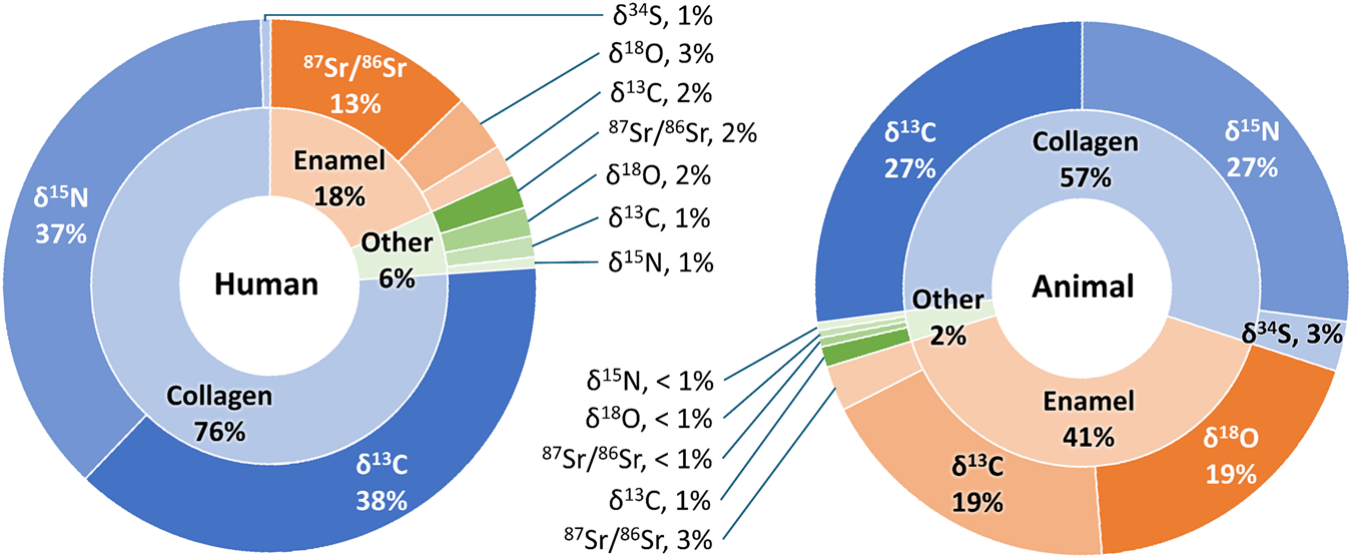
Distribution of the human and animal stable isotopic measurements in NEENA by isotope type and analysed component.

In total, 18,739 isotopic measurements from >750 sites are included in the dataset. Of them, the majority are δ^13^C collagen measurements (6073 IRMS and AMS measurements, comprising 32% of the dataset) and δ^15^N measurements (6009, 32% of the dataset); carbonate measurements form the two next biggest groups with 2011 δ^13^C measurements (11% of the dataset) and 2148 δ^18^O measurements (11% of the dataset); the smallest groups are ^87^Sr/^86^Sr (1711, 9% of the dataset), δ^34^S collagen measurements (337, 2% of the dataset), and δ^18^O phosphate measurements (112, 1% of the dataset) (Tables 1–3, Fig. 3). Although the absolute majority of the records pertain to archaeological samples, several measurements on modern non-archaeological samples were also included where reported in the source publications (65 animal records and 1 plant record).

**Table 1:** Number of isotopic measurements in the NEENA dataset of humans by tissue (“Analysed Component”) and measurement type.

Analysed Component	δ^13^C	δ^15^N	δ^34^S	δ^18^O	^87^Sr/^86^Sr	Total
**Collagen**	3598	3519	59			**7176**
**Bioapatite (non-enamel)**	55			48	122	**225**
**Enamel**	179			328	1197	**1704**
**Keratin**	30	30				**60**
Other*	34	32		116	75	**257**
**Total**	**3896**	**3581**	**59**	**492**	**1394**	**9422**

*‘Other’ represents cases where the source publication did not clearly specify which component the measurement was made on or the measurement was made on an unconventional component, such as bulk bone or dentine, rather than collagen or bioapatite.

**Table 2:** Number of isotopic measurements in the NEENA dataset of animals by tissue (“Analysed Component”) and measurement type.

Analysed Component	δ^13^C	δ^15^N	δ^34^S	δ^18^O	^87^Sr/^86^Sr	Total
**Collagen**	2475	2490	278			**5243**
**Bioapatite (non-enamel)**	59			10	37	**106**
**Enamel**	1718			1725	250	**3693**
**Keratin**	41	41				**82**
Other*	17			33	12	**62**
**Total**	**4310**	**2531**	**278**	**1768**	**299**	**9186**

*‘Other’ represents cases where the source publication did not clearly specify which component the measurement was made on; the measurement was made on an unconventional component, such as bulk bone or dentine, rather than collagen or bioapatite; or a component that appears in the dataset very rarely, such as calcite or calcium carbonate.

**Table 3:** Number of isotopic measurements in the NEENA dataset of plants by sampled element and measurement type.

Sampled Element	δ^13^C	δ^15^N	^87^Sr/^86^Sr	Total
**Charcoal**	1	1		**2**
**Charred grain**	10	10	14	**34**
**Charred wood**			4	**4**
**Fruit stone**	4	4		**8**
**Seed**	7	6		**13**
**Stem**	2	2		**4**
**Wood**	5	2		**7**
Other*	30	29		**59**
**Total**	**59**	**54**	**18**	**131**

Columns for sulfur and oxygen measurements are not included, as no measurements of these isotopes on plants were found.

*‘Other’ represents cases where the source publication did not clearly specify the sampled element.

Among the bioarchaeological remains included in the dataset, 9,422 measurements are on samples from human individuals, and 9,186 are on samples from other animal species. In contrast to these two groups, isotopic measurements of plants are rare in the literature - 131 data points were collected from the reviewed sources. Bulk measurements on collagen, bioapatite, and other organic remains, and incremental measurements from tooth dentine and enamel were included.

The data stored in NEENA are divided into the following broad categories:

- ID Information: unique identifiers for each record, such as NEENA’s internal entry ID, sample and individual identifiers provided in the source publication, and ID of the archaeological context.
- Biological Information (for humans and animals): information on the biological sex and age of humans and other animals.
- Taxonomic Information (for animals and plants): family, genus, and species of animals and plants, accompanied with information about domestication status, living environment and trophic category of animals, and photosynthetic pathway of plants.
- Sample Information: indicates which element of a plant, animal, or human was sampled, including the analysed component (e.g., for humans and animals: collagen, bioapatite, or other).
- Site Information: site name, description and location, including geographic coordinates (decimal latitude and longitude relative to the WGS84 system), the name of the modern country in which the site is situated, and its contemporary cultural or political affiliation.
- Chronological Information: information about the relative and absolute dating of the sample or its context, including range in years ago or years BCE-CE, radiocarbon measurements (where available), and descriptive chronological periods.
- Social information (for humans): where indicated in the source publications, information about the social status, possible occupation, and religious denomination of the sampled human individual were recorded.
- Bibliographic Information: reference (in Harvard citation style) to the source from which the isotopic measurement was collected, including a web link and digital object identifier (DOI). Where the record was sourced indirectly (e.g., from another database of isotopic measurements or using a publication that cited another source that was inaccessible), another set of bibliographic information referencing the sourced compilation was added.
- Isotopic Information (with Collagen and Bioapatite/Enamel sections in the human and animal tables, and a single Organic Plant Remains section in the plant table): the actual isotopic measurement accompanied with information about the lab where it was carried out, the number of samples it represents (some measurements are published only as population means), and the quality parameters such as collagen yield or elemental atomic ratios.
- Notes: the last column in each tab contains free-text notes about the record. Any additional information about the entry that did not fit in other columns was recorded there, such as observations made by the compilers about useful points from the source publications or other literature discussing the site or samples, any uncertainties in the source publications, and assumptions and decisions that had to be made when populating the data. For the best experience with the dataset it is advisable that users always check whether any notes are associated with the entries of interest, as they may contain useful additional details.

A detailed explanation about how each variable was recorded and normalized is provided in the ‘Metadata’ sheet in the dataset files. Three of the variables require additional commentary to clarify the logic used to populate them:

1. Individual ID was designed to be unique in NEENA, therefore an attempt was made to also resolve cases where Individual ID reported in publications were ambiguous or inconsistent. In such cases it was not always clear whether different elements of one individual had been analysed in different studies or whether isotope measurements had been made on multiple individuals from the same context. Only where context descriptions clearly detailed the recovery of a single individual was this assumed (for example, entries NEENAH004680 and NEENAH004699). Where this was not certain Individual IDs were not assigned (for example, entries NEENAH004267 and NEENAH004268). Where it was not certain in a publication that the same individual was being referred to in different places (e.g., in a table and in the text) but it was likely the case, a note was added to the dataset indicating this (e.g., entry NEENAH004230). Where a duplicate measurement was given (i.e., on the same sample, in the same instrument run) an individual ID was not assigned, and such measurements were reported as an average, recorded in one row (for example, entry NEENAH001354). The maximum number of human and animal individuals in NEENA is 6,542: however, the actual number may be smaller.
2. As Sample ID was also meant to be unique, if the same Sample ID was used in more than one publication for different samples the ID was revised so that it is unique (e.g., by adding a postfix such as -col or -en to differentiate collagen and enamel samples of an individual which were both reported with the same ID^67,68^).
3. The uncertainty radius was estimated taking account of all of the information provided about the site location in the source publication or other relevant publications. For example, the site location uncertainty radius could be an average distance to nearby villages for a site named after a modern village (assuming the site would take the name of another village if it were closer to them^19^). For a site located in a city and provided with a street name, the radius would be smaller, in the range of several hundred metres^11,67^. For locations given only through description or low-resolution maps, the uncertainty radius could span to a region of a given country; or to the size of the whole country in the rare cases where only the country name accompanied e.g., animal remains. For example, coordinates given for the site Olenii in the source publication^69^ placed it in Bulgaria instead of Russia, while the site was described to be in Krasnodar Province, in the Kalininskii region of Russia. Since no other publication for this site could be found and it could not be located in Google maps by its name and description, coordinates of, and a radius encompassing, the whole Kalininskii region were used (45.63, 38.39; 44 km).

## Technical Validation

Values of the quality control parameters for collagen preservation (“%C”; “%N”; “Atomic C:N ratio”; “Atomic C:S ratio”; “Atomic N:S ratio”)^13,70,71^ were included where they were provided in the source publication. Both records that met and did not meet these parameters were included, since ‘failed’ measurements may be useful for studying relative preservation and the diagenetic factors that influence it; moreover, different studies may choose to apply different cut-off points for considering a measurement successful or failed, and the acceptable ranges of existing quality parameters may be reconsidered with time.

Studies were included in the dataset regardless of whether quality controls were reported or not. Users of the dataset may choose to filter out those where no values of quality control parameters are reported or refer to the source publication for any additional, relevant details.

Measurements of oxygen and strontium isotope ratios are generally performed on the bioapatite of tooth enamel or bone. Of the two, enamel data is considered the more reliable, due to being less prone to diagenetic alteration^2,72–74^. Measurements on bioapatite from both components were recorded in NEENA, and again users may wish to filter measurements using the ‘Analysed Component’ variable.

Oxygen isotope ratios are typically measured on carbonate or phosphate with the value of both measurements reported calibrated to either the Vienna Pee Dee Belemnite (VPDB) or Vienna Standard Mean Ocean Water (VSMOW) standards; they may also be converted to δ^18^O drinking water^2,74^. Data reported to each of these standards are presented in separate columns in NEENA (e.g., δ^18^O Carbonate (VSMOW); δ^18^O Phosphate (VPDB)). Where a source publication included original and converted values, the original measurements were presented.

## Usage Notes

The NEENA dataset^64^ combines published measurements of the isotopes most commonly measured in archaeological studies (δ^13^C, δ^15^N, δ^34^S, δ^18^O, and ^87^Sr/^86^Sr) with geospatial, chronological, archaeological, historical information, and bibliographic information relating to each record. Such a combination should facilitate regional and inter-regional, as well as site or time-period specific analyses of the Baltic, Eastern Europe, and North-Central Asia, from 70,000 years ago to modern times. In addition, this compilation supports general exploration of isotopic data coverage and gaps, as well as detailing relevant publications. Potential uses include research related to the spread of animal and plant domestication; exploration of aspects of past human lives, such as diet, mobility, and hunter-fisher-gatherer, pastoralist, and agriculturalist subsistence strategies; lives in urban and rural settlements; palaeoenvironmental and palaeoclimatic studies; research into taphonomic factors affecting sample preservation; and freshwater reservoir effects^75–182^.

## Data Availability

The database is deposited within the ‘NEENA Stable Isotopes v.1’ repository^64^ with 10.48493/jgfb-5309, within the ‘NEENA’ data community (https://pandoradata.earth/organization/neena) of the Pandora data platform (https://pandoradata.earth/).

## Code availability

No custom code was used to generate or process the data.
